# Models to predict relapse in psychosis: A systematic review

**DOI:** 10.1371/journal.pone.0183998

**Published:** 2017-09-21

**Authors:** Sarah Sullivan, Kate Northstone, Caroline Gadd, Julian Walker, Ruta Margelyte, Alison Richards, Penny Whiting

**Affiliations:** 1 NIHR CLAHRC West, United Hospitals Bristol NHS Foundation Trust, Bristol, United Kingdom; 2 School of Social and Community Medicine, University of Bristol, Bristol, United Kingdom; 3 Otsuka Pharmaceutical Europe Ltd, trading as Otsuka Health Solutions, Wexham Springs, Slough, United Kingdom; 4 Avon & Wiltshire Mental Health NHS Trust, Jenner House, Chippenham, Wilts, United Kingdom; University of Exeter, UNITED KINGDOM

## Abstract

**Background:**

There is little evidence on the accuracy of psychosis relapse prediction models. Our objective was to undertake a systematic review of relapse prediction models in psychosis.

**Method:**

We conducted a literature search including studies that developed and/or validated psychosis relapse prediction models, with or without external model validation. Models had to target people with psychosis and predict relapse. The key databases searched were; Embase, Medline, Medline In-Process Citations & Daily Update, PsychINFO, BIOSIS Citation Index, CINAHL, and Science Citation Index, from inception to September 2016. Prediction modelling studies were assessed for risk of bias and applicability using the PROBAST tool.

**Results:**

There were two eligible studies, which included 33,088 participants. One developed a model using prodromal symptoms and illness-related variables, which explained 14% of relapse variance but was at high risk of bias. The second developed a model using administrative data which was moderately discriminative (C = 0.631) and associated with relapse (OR 1.11 95% CI 1.10, 1.12) and achieved moderately discriminative capacity when validated (C = 0.630). The risk of bias was low.

**Conclusions:**

Due to a lack of high quality evidence it is not possible to make any specific recommendations about the predictors that should be included in a prognostic model for relapse. For instance, it is unclear whether prodromal symptoms are useful for predicting relapse. The use of routine data to develop prediction models may be a more promising approach, although we could not empirically compare the two included studies.

## Introduction

People with psychosis have a high likelihood of relapse. The cumulative relapse rate five years after initial recovery from psychosis is 82% and the second relapse rate is 78% [1]. Relapses cause distress for patients and their carers [2]. It has also been suggested that repeated relapses may have an adverse effect on the brain in terms of cognitive deterioration and less complete recovery from subsequent relapses [3]. There is a reported association between relapse and reduced social functioning, unemployment and social isolation [4] and evidence of a dose-response effect with repeated relapses associated with greater cognitive decline and poorer social functioning [3]. A recent projection of the total expenses of schizophrenia in the UK reported costs of £1 billion per year [5], a significant proportion of which is inpatient treatment [6], which may be a consequence of the most serious relapses. There is some evidence from a recent systematic review to suggest that it is possible to intervene to reduce the likelihood of relapse [7]. The most successful interventions reported were: patient psycho-education, structured needs assessments, medication reconciliation and education, transition managers and inpatient/outpatient provider communication. A tool to predict relapses in people with psychosis could improve patient-outcomes, inform therapeutic decision-making, allow the appropriate targeting of mental health service resources and therefore reduce treatment costs. Evidence in favour of the accuracy of a tool to predict relapse in psychosis would therefore be advantageous both for mental health care providers as well as service users and carers. We are not aware of any systematic review of relapse prediction tools in psychosis. Our aim was to systematically review the literature on existing models to predict relapse in people with psychosis.

## Method

This review followed the guidance published by the Centre for Reviews and Dissemination [8] and the Cochrane Prognosis Methods Group [9]. We established a protocol for the review (S1 File) which pre-specified objectives, eligibility criteria and review methods. Reporting of the review followed the PRISMA checklist (S1 Table).

### Identification of studies

Seven electronic databases: Embase (OvidSP), Medline (OvidSP), Medline In-Process Citations & Daily Update (OvidSP), PsychINFO (OvidSP), BIOSIS Citation Index (Web of Science), CINAHL (Cumulative Index to Nursing and Allied Health Literature–EBSCO), and Science Citation Index (Web of Science) were searched from inception to September 2016 to identify relevant studies of clinical prediction models of relapse in psychosis. Search methods met best practice standards in systematic reviews [8, 10]. The search strategy (S2 File) combined terms for psychosis and relapse with the Ingui filter for identifying prediction modelling studies [11]. Searches were not limited by language, date or publication status.

An internet search using the Google search engine and screening reference lists of included studies were used to identify any additional relevant unpublished studies (grey literature). The authors of any grey literature were contacted to find out whether there were any unpublished study results available.

### Study selection

The inclusion criteria were defined based on the CHARMS (**Ch**ecklist for critical **A**ppraisal and data extraction for systematic **R**eviews of prediction **M**odelling **S**tudies) [12] guidelines. We included studies that described prediction model development with or without external model validation studies. To qualify as a prediction modelling study a paper must have reported a full multivariate model including regression coefficients and formally presented a model that could be used to predict the probability of a psychotic relapse. Models had to target people with a psychotic disorder (including schizophrenia), with single and multiple psychotic episodes, and predict relapse or repeated relapse defined as admission or readmission to a psychiatric inpatient unit or recurrence of psychotic symptoms over a threshold level (as defined in the included study). There was no restriction on the time span of prediction or the intended moment of using the model. Search results and full text articles were independently assessed by two reviewers; disagreements were resolved through discussion or referral to a third reviewer.

### Data extraction

Data were extracted using standardised data extraction forms developed in Microsoft Access 2010. The forms were initially piloted on a small sample of papers and adapted as necessary. To minimise bias and errors, data extraction was performed by one reviewer and checked by a second. Data extracted for each study included; country of study, funding source, potential conflicts of interest, type of study, participants, type of prediction model, duration of model testing, types of outcome measure and types of predictors included in the model.

### Quality assessment

Prediction modelling studies were assessed for risk of bias and applicability using the PROBAST tool [13]. The assessment of risk of bias includes the domains of participant selection, outcome, predictors, sample size and flow, and analysis. The first three domains are also assessed for applicability to the systematic review question.

We used the PROBAST tool guidance to reach an overall judgement of risk of bias. This stated that even if all domains were rated at low risk of bias a downgrade to a high risk should be considered without validation of the model developed. A rating of low risk of bias should only be considered if the development was based on a very large dataset and there was some form of internal validation. The risk of bias assessment was conducted as part of the data extraction process.

### Analysis

Due to the small number of included studies and differences between studies meta-analysis was not appropriate. A narrative synthesis was performed, including a summary of study characteristics (study design, population size, geographical location, year, baseline population characteristics, outcome definition and assessments) and findings reported as descriptive text and tables. A detailed commentary on major methodological problems and biases was also included.

## Results

**Fig 1:**
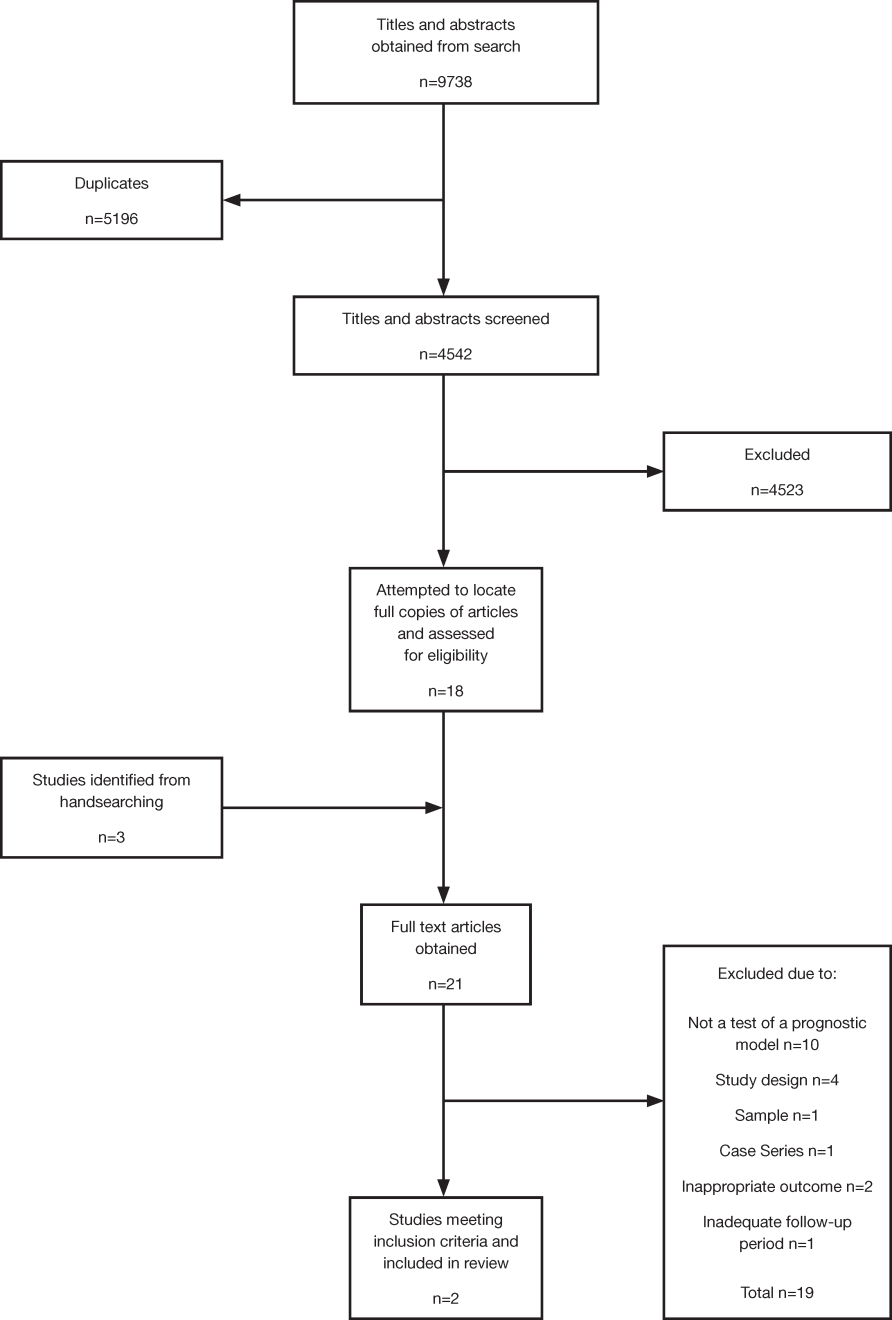
PRISMA Flowchart of search and review strategy.

The search identified 9,838 hits of which two studies (total participants = 33,088) [14, 15] met inclusion criteria (Fig 1). An additional six ongoing pilot studies from the grey literature search were identified. After contact with the investigators listed in the grey literature it was found that results from these studies were not available at the time of enquiring. These unpublished studies were all testing methods to predict relapse using personal technologies such as smart phone apps or wrist-worn activity monitors [16–21].

The included studies were published in 2007 and 2015 and were set in Germany and Canada. One [14] was restricted to people with a diagnosis of schizophrenia and used participants from a trial of medication discontinuation and the second [15] included a broader diagnostic category of psychotic disorder. Mean age was 39 years. Both were cohort studies and had samples with slightly more women and included patients who had suffered multiple psychotic episodes with illness durations of five to seven years. One study reported model development only and one [15] developed and validated a model using a split data sample method. Tables 1 and 2 provide an overview of the included studies.

**Table 1:** Studies included in systematic review.

Study and location	Source of Data	Sample size	Participants	Diagnosis	Outcome	Candidate Predictors	Final Model Predictors
Gaebel et al 2007 [14] Germany	Cohort follow up over 2 years. Participants drawn from a randomised controlled trial on medication discontinuation.	339	46.3% male. Mean age 43.8 (9.3) years. Illness duration 7.2 (7.5) years. 31% first episode. Recruited from outpatient clinics.	Schizophrenia	Clinically: psychotic deterioration usually requiring hospitalisation. A change in 3 objective measures; BPRS^a^ ≥10, CGI^b^-Change ≥6, decrease GAS^c^≥20. Mean time between assessments 28 days. 227 relapses in 153 patients recorded.	1/ Six prodromal symptoms from Early Symptom Questionnaire; tense and nervous, depression, trouble sleeping, restlessness, trouble concentrating, loss of interests. 4 point Likert scale and dichotomised (0 vs ≥1). Mean time between assessments 28 days 2/ Up to 3 of 16 additional prodromal symptoms which predicted relapse and selected individually at study entry. 4 point Likert scale and dichotomised (0 vs ≥1). Mean time between assessments 28 days 3/ Overall prodrome score created from score of each symptom (0–27), also dichotomised using different cut offs. 4/ Time between prodromal symptom report and relapse (dichotomised <21 days vs ≥21 days). 5/ Psychopathology (BPRS factors) 6/ CGI items severity and change 7/ GAS (contact prior to predicted event) 8/ cumulative dose of neuroleptic medication 9/ treatment group from trial	1/ Single prodromal symptom ‘trouble sleeping’. 2/ Overall prodromal score (OPS) 3/ BPRS items: depression, suspiciousness and BPRS factor motor-retardation. 4/ CGI change 5/ Treatment group: crisis intervention and prodrome based early intervention.
Vigod et al 2015 [15] Canada	Routine data	Total sample n = 65,789 Model derivation sample n = 32,749 Model validation sample n = 33040	49.2% male. Mean age 42.5 years, 88.6% urban community, 28.7% lowest neighbourhood income quintile, 26.2% employed, 32.6% married/partner, homeless 2%, ≤high school 50.2%.	Psychotic disorder	Psychiatric readmission to any hospital within 30 days of discharge from index admission	Sociodemographic variables (age, sex, community size, marital status, living situation, type of residence, source of income, educational attainment) prior healthcare utilisation (number of previous psychiatric hospital admissions within 2 years before index admission and number of psychiatric emergency department and outpatient mental health visits within 1 year before admission and overall medical comorbidity) basic clinical and administrative information from a hospital admission (criteria met for involuntary admission, harm to self or others, inability to care for self, length of stay, planned or unplanned discharge, psychiatric diagnoses, discharge GAF score) and detailed psychiatric rating scales and metrics administered by clinicians during an admission (symptom, functional and behavioural domains).	Risk index created from: Number of prior admissions, harm to self, harm to others, inability to care for self, age, diagnosis of psychosis, bipolar and personality disorder, unplanned discharge, medical comorbidity, intensity of outpatient and emergency department use prior to admission and time in hospital. Score ranged from 0–41

Key:

^a^ Brief Psychiatric Rating Scale

^b^ Clinical Global Impression

^c^ Global Assessment Scale

**Table 2:** Type of study and findings.

Study	Model development	Model classification	Model performance	Model validation
Gaebel et al 2007 [14]	Sensitivity, specificity, ORs^a^. Logistic regression also with additional variables. ROC^b^ analysis with different cut offs for the overall prodromal score.	Development only	Trouble sleeping OR^a^ 1.42 p = 0.05 and overall prodromal score OR^a^ 1.03 p = 0.003. Total r squared from final model 0.14 –of limited predictive value. AUC^c^ = 0.59 p<0.59.	N/A
Vigod et al 2015 [15]	Split sample. Model built in one half and validated in the other half. Four models tested. Model Derivation Model 1 –sociodemographic variables only; Model 2 Model 1 + healthcare utilisation; Model 3 Model 2 + clinical and administrative admission information; Model 4 Model 3 + detailed rating scales. Log likelihood test to test for improvement in prediction as each model added. Multivariable logistic regression used. Model fit assessed using Hosmer-Lemeshow goodness of fit and discrimination using C statistic Creation and validation of risk index Risk index system developed from above. Probability of 30 day readmission calculated for each score of the risk index. C statistic calculated for derivation and validation samples	Development and validation	Association between risk index and outcome OR^a^ 1.11 (95% CI 1.10–1.12). Probability of 30-day readmission using risk score from 2% at score of 0 to 49% at a score of 41. Probability of readmission was within the 95% CI of the observed probability for all scores in derivation and validation sample, indicating adequate calibration. Model performance described as moderate for derivation (C statistic = 0.631) and validation (C statistic = 0.630).	Validated in 32,750

Key

^a^ Odds Ratio

^b^ Receiver Operating Curve

^c^ Area under the Curve

Relapse was defined as an exacerbation of psychotic symptoms and a decrease in functioning [14] or as hospitalisation [15].

The number of candidate predictors used to develop the prediction models were 9 [14] and 22 [15]. The predictors were prodromal symptoms and global functioning [14]. and population-based health administration data [15] (socio-demographic variables, prior health service use, medical comorbidity, clinical and administrative information collected during index admission and detailed rating scales and metrics calculated at discharge and admission).

Methods used to select candidate predictors for inclusion in the final model differed. One study [14] used analyses based on 2x2 tables i.e. prodrome yes/no with relapse yes/no to derive measures of sensitivity (the percentage of relapses correctly identified by the model–true positives) and specificity (the percentage of non-relapses correctly identified by the model–true negatives). Sensitivity and specificity was calculated for various cut-offs using a Receiver Operating Characteristics (ROC) analysis. Coefficients from logistic regression models were used to select variables from the candidate predictors for the final prediction model. The other study[15] used log likelihood and Chi square tests to make decisions about including predictors in the model. (see Table 2).

Multivariable logistic regression was used to fit the most predictive model in both studies. Overall model fit was examined as well as discrimination using a C test in the study which validated its model [15]. To do this, the final logistic regression model was converted into a risk index and a probability of relapse was created for each score of the risk index. A C statistic and expected and observed probabilities of relapse were generated for both the derivation and validation samples to determine the calibration of the risk index.

One study [14] was judged at high risk of bias and the other [15] at low risk of bias. The high risk of bias rating was due to concerns over the assessment of predictors and outcomes. Other areas of concern were that not all the participants were included in the analysis and the model developed was not validated. In contrast, the study rated at low risk of bias [15] had no major areas of concern, largely because of the use of routine data sources. This study developed and validated a prediction model using a split sample method, which is a less rigorous method of model validation than using external data. An overall low risk of bias was considered appropriate for this study however, because model development was carried out on an extremely large dataset and there was internal model validation. This rating is recommended under these circumstances by the PROBAST tool [13]. See Table 3 for bias assessment results.

**Table 3:** Quality assessment of studies included in the systematic review–areas of concern using PROBAST guidelines.

Study	Domain 1: Participant selection		Domain 2: Predictors		Domain 3: Outcomes		Domain 4: Sample size and participant flow	Domain 5: Analysis	Overall Judgement
	Risk of Bias	Applicability	Risk of Bias	Applicability	Risk of Bias	Applicability	Risk of Bias	Risk of Bias	
Gaebel et al 2007 [14]	Low Justification Participants not in similar state of health but other predictors such as psychopathology were included in to model to adjust for this.	Low Justification Participants, setting and dates match review question.	High Justifications 1/ Assessors of predictors not blinded to outcome data. 2/ Predictors were not defined in same way for all participants.	Low Justification Definition, assessment and timing of assessments match review question.	High Justification Assessors of outcome not blinded to predictor data.	Low Justification Definition, timing and determination match review question.	High Justification Not all participants included in analysis and not otherwise accounted for.	Low Justification Participants, setting and dates match review question.	High Justification At least one domain at high risk of bias
Vigod et al 2015 [15]	Low Justification No concerns	Low Justification Participants, setting and dates match review question.	Low Justification No concerns	Low Justification Definition, assessment and timing of assessments match review question. .	Low Justification No concerns	Low Justification Definition, timing and determination match review question.	Low Justification No concerns	Low Justification Split sample a less rigorous method of model validation BUT model was based on a very large dataset and was internally validated.	Low Justification All domains at low risk of bias and internal validation concerns reduced by use of very large dataset.

One study [15] had a short (i.e. 30 day) follow up period because its aim was to predict early relapse. The other [14] had a longer follow up period (i.e. 2 years). The short follow up periods of the included studies potentially reduced the number of relapses and included a higher proportion of relapses occurring soon after recovery, which are likely to occur in those who are more unwell or who are suffering from residual symptoms and who therefore may be more likely to relapse. The findings therefore may not be generalizable to those who have recovered from psychosis without residual symptoms.

There were no concerns regarding applicability for either of the included studies.

### Study findings

The sensitivity of individual prodromal symptoms was below 40% and specificities ranged from 70% to 95% [14]. The sensitivity for the overall prodromal score (OPS) at different cut offs ranged from 25% (specificity = 86%) to 72% (specificity 38%) [14]. The optimum cut-off for the OPS score was ≤3 (no prodromal state) vs ≥4 (prodromal state) with a sensitivity of 39%, a specificity of 76%. The Area under the Curve (AUC) statistic was 0.59 The model which included time between symptom report and relapse increased sensitivity to 80% if the time-period was <21 days. Only depression, suspiciousness, motor-retardation, change in CGI score were significantly associated with the outcome. The final prediction model (i.e. the OPS, depression, suspiciousness, motor-retardation, change in CGI score, treatment group and a single prodromal symptom “trouble sleeping”) only explained 14% of the variance in outcome.

The study [15] which used administrative data to form a risk of relapse prediction index reported that a model containing data on socio-demographics, prior health service and clinical and administrative variables was the most predictive model. The variables included in the final model are shown in Table 2. The risk index (created from the final logistic regression model) was associated with the outcome (OR 1.11 95% CI 1.10, 1.12) and the association appeared to be linear. The model indicated acceptable calibration (C = 0.631 for the development dataset and C = 0.630 for the validation dataset).

## Discussion

### Summary of results

We identified two studies that assessed the accuracy of models to predict relapse in people with psychosis. One model was of limited predictive value [14] and the other [15] had moderate discriminatory power.

One [14] of the studies was judged to be at high risk of bias, which may have resulted an overestimate of the association. The second study [15] was judged at low risk of bias, in spite of the fact that internal model validation was used, because it was conducted in a very large dataset.

### Comparison with previous studies

We are not aware of any previous systematic reviews on prediction models for relapse in psychosis. However, there is one related systematic reviews of risk factors for relapse [22], which included 29 references and conducted a meta-analysis of 20 predictors. Medication non-adherence, persistent substance abuse, carers’ critical comments and poor pre-morbid adjustment predicted the risk of relapse by between 2.2 to 4-fold. There are also two systematic reviews and meta-analyses of randomised controlled trials of second-generation versus first-generation antipsychotics for reducing relapse in psychosis[23, 24]. Both these reviews found that second-generation antipsychotics were more effective at reducing relapse. Finally, there is one systematic review of transitional interventions to reduce early psychiatric readmissions [7]. Fifteen studies were reviewed and five successful interventions were identified (psychoeducation, structured needs assessments, medication education, transition managers and communication between service providers. It is interesting to notes that neither of the prediction models reported here included any of these variables.

### Strengths and limitations

This systematic review has been conducted using validated and robust methods and the quality of the studies was evaluated using PROBAST, a new tool that has been developed by methodological experts in the area of clinical prediction tools and quality assessment [13].

The small number of studies identified in the review, as well as the differences in the measures of relapse and predictors assessed, meant that it was not appropriate to conduct a formal meta-analysis. One the most important differences between the studies included our review was the use of different measures to define a relapse. One study [14] defined relapse as a pre-defined change in psychometric measures of symptoms, severity and functioning and the other [15] used admission to hospital. Future studies in this area should move towards a uniform measure of relapse to facilitate the pooling of findings. This inconsistency has made it difficult to compare study findings.

The mean age of the participants in the included studies suggests that the results are more applicable to older people with chronic psychosis rather than younger people who have been newly diagnosed and are experiencing their first episode of psychosis.

### Clinical implications

Because our systematic review only found two relevant studies which each used a different set of predictors and one of which was at high risk of bias, it is not possible to recommend either of these methods of predicting relapse in psychosis. For instance, it is not yet clear whether the emergence or worsening of prodromal symptoms can accurately predict an impending psychotic relapse. It is possible that a more promising approach may be the use of administrative data [15]. However, this finding would require replication and external validation before any conclusion could be reached. The variables used may include a combination of demographic variables such as age, clinical factors such as diagnosis, measures of severity including harm to self and others and inability to care for oneself as well as physical illness comorbidities and data on history of health care service use such as intensity of outpatient and emergency service use. An important advantage to this approach is that administrative data is readily available and therefore avoids the extra cost and effort of collecting additional prodromal and psychotic symptom data. However, it is also important to consider the well-publicised problems with administrative data, such as unexplained missingness and poor quality, such as recording errors.

The ability to accurately predict a psychotic relapse would represent an important step forward in mental health care. It would be particularly useful for mental health system leadership teams to make decisions on the appropriate use of resources, particularly in an environment when such resources are in short supply. Those who are at risk of crisis could be allocated more intensive care with experienced clinicians, whereas those at a lower risk could be allocated to less intensive or a step-down of care. Resource planning is difficult and time-consuming without such information. It is also possible that accurate prediction of which service users were most likely to relapse may allow targeting of an intervention to reduce the probability of a relapse. This may take the form of increasing medication dose or psychological intervention. If successful, an intervention which reduces the number of relapses would also be an important development since there is evidence that a reduction in the number of psychotic episodes is associated with better outcomes and reduced distress to the service user and their carer [2, 3, 25, 26]. There would also be the advantage of reduced treatment costs arising from reduced inpatient admissions because of a relapse. There is also some evidence [27] that service users could be taught to self-identify relapse indicators and therefore self-manage prodromal symptoms in order to reduce the probability of relapse. It has also been suggested [7] that clinicians could perform an unmet needs assessment for service users predicted to be at high risk of relapse and that such assessments can greatly reduce the risk of psychiatric readmission.

### Future research

It is possible that a prediction model based on administrative data may be a useful approach, although this needs to be replicated and validated in further datasets. The approach based on prodromal symptoms may be less useful but to be sure the study should be repeated in a larger sample with a comparison group and a longer follow-up period. Attention should also be paid to some of the methodological issues highlighted in this review. For example, it would be preferable to measure relapse and predictors on different occasions and to ensure that the outcome assessor is blinded to the predictor assessment. Studies which have investigated sensitivity and specificity of prodromal symptoms may also uncover useful information for further model development with an investigation of relapses that are predicted by the model but do not occur.

## Supporting information

S1 File Systematic review protocol. (DOCX)

S2 File Search terms. (DOCX)

S1 Table PRISMA 2009 checklist. (DOC)

## References

[1] Robinson D, Woerner MG, Alvir JM, Bilder R, Goldman R, Geisler S, et al Predictors of relapse following response from a first episode of schizophrenia or schizoaffective disorder. Archives of general psychiatry. 1999;56(3):241–7. . 10078501 10.1001/archpsyc.56.3.241

[2] Weiden PJ, Olfson M. Cost of Relapse in Schizophrenia. Schizophrenia Bulletin. 1995;21(3):419–29. 7481573 10.1093/schbul/21.3.419

[3] Wiersma D, Nienhuis FJ, Slooff CJ, Giel R. Natural course of Schizophrenic disorders: A 15-year followup of a Dutch incidence cohort. Schizophrenia Bulletin. 1998;24(1):75–85. 9502547 10.1093/oxfordjournals.schbul.a033315

[4] Rooke O, Birchwood M. Loss, humiliation and entrapment as appraisals of schizophrenic illness: A prospective study of depressed and non-depressed patients. Br J Clin Psychol. 1998;37:259–68. 9874588 10.1111/j.2044-8260.1998.tb01384.x

[5] Schizophrenia Commission. The Abandoned Illness. 2012.

[6] Hong JY, Windmeijer F, Novick D, Haro JM, Brown J. The cost of relapse in patients with schizophrenia in the European SOHO (Schizophrenia Outpatient Health Outcomes) study. Progress in Neuro-Psychopharmacology & Biological Psychiatry. 2009;33(5):835–41. doi: 10.1016/j.pnpbp.2009.03.034. 19351551 10.1016/j.pnpbp.2009.03.034

[7] Vigod SN, Kurdyak PA, Dennis CL, Leszcz T, Taylor VH, Blumberger DM, et al Transitional interventions to reduce early psychiatric readmissions in adults: systematic review. British Journal of Psychiatry. 2013;202(3):187–94. doi: 10.1192/bjp.bp.112.115030. 23457182 10.1192/bjp.bp.112.115030

[8] Centre for Reviews and Dissemination. Systematic Reviews: CRD’s guidance for undertaking reviews in health care York: University of York, 2009.

[9] Collaboration. C. Cochrane Methods Prognosis. Trusted evidence. Informed decisions. Better health. 2016. Available from: http://methods.cochrane.org/prognosis.

[10] Higgins JPT, Green S, editors. Cochrane handbook for systematic reviews of interventions [Internet]. Version 5.1.0 [updated March 2011]: The Cochrane Collaboration; 2011 [accessed 23.3.11].

[11] Geersing G-J, Bouwmeester W, Zuithoff P, Spijker R, Leeflang M, Moons KGM, et al Search filters for finding prognostic and diagnostic prediction studies in Medline to enhance systematic reviews. PLoS One. 2012;7(2):e32844 doi: 10.1371/journal.pone.0032844 . 22393453 10.1371/journal.pone.0032844 PMC3290602

[12] Moons KG, de Groot JAH, Bouwmeester W, Vergouwe Y, Mallett S, Altman DG, et al Critical appraisal and data extraction for systematic reviews of predictgion modelling studies: The CHARMS Checklist. PLoS One. 2014;11(10). 10.1371/journal.pmed.1001744 PMC4196729 25314315

[13] Wolff R, Whiting P, Westwood M, Kleijnen J, Mallet S, Riley R, et al. PROBAST 2015. Available from: http://www.systematic-reviews.com/.

[14] Gaebel W, Riesbeck M. Revisiting the relapse predictive validity of prodromal symptoms in schizophrenia. Schizophrenia Research. 2007;95(1–3):19–29. doi: 10.1016/j.schres.2007.06.016 . 17630253 10.1016/j.schres.2007.06.016

[15] Vigod SN, Kurdyak PA, Seitz D, Herrmann N, Fung K, Lin E, et al READMIT: a clinical risk index to predict 30-day readmission after discharge from acute psychiatric units. Journal of Psychiatric Research. 2015;61:205–13. http://dx.doi.org/10.1016/j.jpsychires.2014.12.003. doi: 10.1016/j.jpsychires.2014.12.003 . 25537450 10.1016/j.jpsychires.2014.12.003

[16] Dawson M. Preventing relapse amongst schizophrenia patients: University of Southern California; 2013 Available from: https://news.usc.edu/56486/preventing-relapse-among-schizophrenia-patients/.

[17] Dobson R, MacCabe J. Sleep sensors to prevent relapse in schizophrenia 2014. Available from: http://www.kcl.ac.uk/ioppn/news/records/2014/October/Sleep-sensors-to-detect-relapse-in-schizophrenia.aspx.

[18] Eisner E, Drake R, Barrowclough C. Assessing early signs of relapse in psychosis: Review and future directions. Clinical Psychology Review. 2013;33(5):637–53. doi: 10.1016/j.cpr.2013.04.001. 23628908 10.1016/j.cpr.2013.04.001

[19] Ben-Zeev R. Smart phone app keeps watch over schizophrenia patients 2014. Available from: http://spectrum.ieee.org/biomedical/diagnostics/smartphone-app-keeps-watch-over-schizophrenic-patients.

[20] Davis UC. App aims to prevent relapse in psychosis patients. 2015.

[21] Lahti AC. Prediction of relapse in schizophrenia/schizoaffective disorder with smartphones and on-body sensors 2014. Available from: https://www.clinicaltrials.gov/ct2/show/NCT02224430.

[22] Alvarez-Jimenez M, Priede A, Hetrick SE, Bendall S, Killackey E, Parker AG, et al Risk factors for relapse following treatment for first episode psychosis: A systematic review and meta-analysis of longitudinal studies. Schizophrenia Research. 2012;139(1–3):116–28. doi: 10.1016/j.schres.2012.05.007. 22658527 10.1016/j.schres.2012.05.007

[23] Kishimoto T, Agarwal V, Kishi T, Leucht S, Kane JM, Correll CU. Relapse prevention in schizophrenia: a systematic review and meta-analysis of second-generation antipsychotics versus first-generation antipsychotics. Molecular Psychiatry. 2013;18(1):53–66. doi: http://dx.doi.org/10.1038/mp.2011.143. doi: 10.1038/mp.2011.143 ; PubMed Central PMCID: PMCNIHMS329515 PMC3320691. 22124274 10.1038/mp.2011.143 PMC3320691

[24] Leucht S, Barnes TR, Kissling W, Engel RR, Correll C, Kane JM. Relapse prevention in schizophrenia with new-generation antipsychotics: a systematic review and exploratory meta-analysis of randomized, controlled trials. American Journal of Psychiatry. 2003;160(7):1209–22. doi: 10.1176/appi.ajp.160.7.1209 . 12832232 10.1176/appi.ajp.160.7.1209

[25] Wiersma D, Wanderling J, Dragomirecka E, Ganev K, Harrison G, an der Heiden W, et al Social disability in schizophrenia: Its development and prediction over 15 years in incidence cohorts in six European centres. European Psychiatry. 2000;15:292s-s. doi: 10.1016/S0924-9338(00)94276-7. ISI:000165731700255. 10.1017/s0033291799002627 12027051

[26] Buchanan RW, Kreyenbuhl J, Kelly DL, Noel JM, Boggs DL, Fischer BA, et al The 2009 Schizophrenia PORT Psychopharmacological Treatment Recommendations and Summary Statements. Schizophrenia Bulletin. 2010;36(1):71–93. doi: 10.1093/schbul/sbp116. 19955390 10.1093/schbul/sbp116 PMC2800144

[27] Tarrier N. Management and modification of residual psychotic symptoms In: Birchwood M, Tarrier N, editors. Innovations in the psychological management of schizophrenia. Chichester: Wiley; 1991.

