# Supplementary material for: Models to predict relapse in psychosis: A systematic review
Source: PLoS One. 2017 Sep 21;12(9):e0183998. doi: 10.1371/journal.pone.0183998 (PMC5608199; doi:10.1371/journal.pone.0183998)
Supplement: S1 File — (DOCX) [file pone.0183998.s001.docx]

CLAHRC West

A systematic review of tools to predict repeated relapse in psychosis

Protocol by NIHR CLAHRC West

20th October 2015

Effectiveness and Evidence Team

NIHR CLAHRC West

University Hospitals Bristol NHS Foundation Trust

9th Floor

Whitefriars

Lewins Mead

Bristol BS1 2NT

http://clahrc-west.nihr.ac.uk/

1. Background

Psychotic illnesses, including schizophrenia, are serious disorders characterised by a loss of contact with reality, psychotic symptoms and poor social functioning. Psychosis is not rare; annual incidence and lifetime prevalence are approximately 0.2 per and 0.4 per 1000 respectively. The most recent data (2004-5) suggest that approximately 300,000 people in the UK have psychosis. The figures for 2015 are likely to be similar. The mean age of onset is approximately 22 years. Psychosis is frequently life-long. The cumulative relapse rate five years after initial recovery is 81.9% and the second relapse rate is 78% {Robinson, 1999}. The World Health Organisation ranks the psychoses as the third most disabling condition worldwide {World Health Organisation, 2004}. A recent projection of the total expenses of schizophrenia in the UK until 2026 reported costs of £6.5 billion. A significant proportion of the costs of the treatment of psychosis is inpatient treatment. A prognostic prediction tool would inform therapeutic decision making, allow the appropriate targeting of mental health services resources to reduce the probability of relapse and therefore reduce treatment costs.

2. Objectives

To review existing prognostic prediction models for relapse to inform therapeutic decision making in patients with psychosis.

3. Methods

3.1 Literature search

Attempts will be made to identify relevant studies of clinical prediction models of relapse in psychosis. Search methods will meet best practice standards in systematic reviews.(1, 2) The following databases will be searched from inception to present:

The following databases will be searched from inception to present:

• Embase (OvidSP): 1974-2015

• Medline (OvidSP): 1946-2015

• Medline In-Process Citations & Daily Update (OvidSP): up to 2015

• PsycINFO (OvidSP): 1806-2015

• BIOSIS Citation Index (Web of Science): 1926-2015

• CINAHL (Cumulative Index to Nursing and Allied Health Literature) (EBSCO): 1982-2015

• Science Citation Index (SCI) (Web of Science): 1900-2015

The search strategy will combine terms for psychosis and relapse with the Ingui filter for identifying prediction modelling studies.(3) A draft Medline search strategy is presented in Appendix 1. The strategy will be adapted to run on the other databases listed. Searches will not be limited by language, date or publication status (unpublished or published).

Handling of citations

Identified references will be downloaded into Endnote X7 software. Individual records within the Endnote reference libraries will be tagged with searching information to enable the information specialist to track the origin of each record.

3.2 Inclusion criteria

The inclusion criteria were defined based on the CHARMS (Checklist for critical Appraisal and data extraction for systematic Reviews of prediction Modelling Studies) (ref Moons et al 2014) guidelines. Studies that fulfil the following criteria will be eligible for inclusion:

Intended scope of the review

Models to inform physicians’ therapeutic decision making

Type of prediction modelling studies

We will include all types of prediction modelling studies: prediction model development with or without external validation in independent data and external model validation studies.

Target Population to whom the prediction model applies

People with a psychotic disorder (which includes schizophrenia), this will include both single and multiple episode populations.

Outcome to be predicted

Relapse or repeated relapse defined as; admission or readmission to a psychiatric inpatient unit, recurrence of psychotic symptoms over a threshold level (as defined in the paper).

Time span of prediction

Any time span for prediction of relapse will be eligible for the review

Intended moment of using the model

Prediction models focussing on a particular moment in time will also be eligible for the review

Existing systematic reviews will be included if they fulfil inclusion criteria and searches were conducted within the past year, otherwise they will be used as a source of potentially relevant studies.

3.3 Methods of study selection, data extraction and quality assessment

Study selection

Titles and abstracts identified through electronic database and web searching will be independently screened by two reviewers. During this initial phase of the screening process any references which obviously do not meet the inclusion criteria will be excluded. Full text copies will be obtained for all remaining references. These will then be independently examined in detail by two reviewers in order to determine whether they meet the criteria for inclusion in the review. All papers excluded at this second stage of the screening process will be documented along with the reasons for exclusion. With respect to both screening stages, any discrepancies between reviewers will be resolved through discussion or the intervention of a third reviewer.

Data extraction

Data will be extracted using standardised data extraction forms developed in Microsoft Access 2010. Data extraction forms will be piloted on a small sample of papers and adapted as necessary. In order to minimise bias and errors, data extraction will be performed by one reviewer and checked by a second. Disagreements will be resolved through discussion or referral to a third reviewer where necessary.

Quality assessment

Systematic reviews will be assessed for methodological quality using the ROBIS tool. (4) This tool aims to assess the risk of bias in systematic reviews and includes domains covering study eligibility criteria, identification and selection of studies, data collection and study appraisal, synthesis and findings, and interpretation.

Prediction modelling studies will be assessed using the PROBAST tool (5) which includes domains covering participant selection, outcome, predictors, sample size and flow, and analysis.

If at least one of the domains is rated as “high” the study will be considered at high risk of bias, if all domains are judged as “low” the trial will be considered at low risk of bias, otherwise the trial will be considered at “unclear” risk of bias. The risk of bias assessment will be conducted as part of the data extraction process.

3.4 ANALYSIS

Narrative synthesis methods

A narrative summary of the included studies will be presented. This will include a summary of the characteristics (e.g. study aim, study design, population size, geographical location, year, baseline population characteristics, outcome definition and assessments). If data are considered too heterogeneous to pool, or not reported in a format suitable for pooling then we will employ a narrative synthesis. This will involve the use of descriptive text and tables to summarise data in order to allow the reader to consider outcomes in the light of differences in study designs and potential sources of bias for each of the studies being reviewed. This involves organising the studies by (as appropriate) population or outcomes assessed, summarising the results of the studies, summarising the range and size of the associations these studies report, and describing the most important characteristics of the included studies. A detailed commentary on the major methodological problems or biases that affected the studies will also be included, together with a description of how this has affected the individual study results.

Quantitative analysis and meta-analysis methods

If sufficient studies assessing similar populations and outcomes are found then a formal meta-analysis will be used to estimate summary measures of effect.

Risk prediction models will be pooled using the methods recommended by Debray et al {Debray, 2014}. We anticipate that systematic differences between studies (heterogeneity) will be likely. Therefore, the random-effects model will be used to calculate summary estimates. Heterogeneity will be investigated visually using forest plots and statistically using the I2 and Q statistics.(6)

A detailed analysis plan will be produced before the analysis is conducted. Statistical analyses will be performed using Stata (version 10) and RevMan (version 5).

4. Timetable

The proposed timeline for the project is summarised below:

Deadline Project stage

7/9/2015 Protocol development

8/9/2015 Finalise protocol

18/9/2015 Searches for studies

2/10/2015 Screening search results

16/10/2015 Inclusion assessment of full papers,

23/10/2015 Data extraction

30/10/2015 Analyses

30/11/2015 Write journal articles

5. Team members

Name Role

Drs Sarah Sullivan/Kate Northstone Project Lead

Dr Sarah Sullivan Lead Reviewer

Dr Kate Northstone Second reviewer

Alison Richards Information specialist

Dr Julian Walker Clinical advice

Dr Will Hall Clinical advice

Dr James Eldred Clinical advice

Dr Penny Whiting Methodological advice

6. References

1. Centre for Reviews and Dissemination. Systematic Reviews: CRD’s guidance for undertaking reviews in health care [Internet]. York: University of York, 2009 [accessed 23.3.11].

2. Higgins JPT, Green S, editors. Cochrane handbook for systematic reviews of interventions [Internet]. Version 5.1.0 [updated March 2011]: The Cochrane Collaboration; 2011 [accessed 23.3.11].

3. Geersing G-J, Bouwmeester W, Zuithoff P, Spijker R, Leeflang M, Moons KGM, et al. Search filters for finding prognostic and diagnostic prediction studies in Medline to enhance systematic reviews. PLoS One. 2012;7(2):e32844.

4. Whiting P, Savovic J, Higgins JP, Caldwell DM, Reeves BC, Shea B, et al. ROBIS: A new tool to assess risk of bias in systematic reviews was developed. Journal of clinical epidemiology. 2015.

5. Wolff R, Whiting P, Westwood M, Kleijnen J, Mallet S, Riley R, et al. PROBAST 2015. Available from: http://www.systematic-reviews.com/.

6. Higgins JP, Thompson SG. Quantifying heterogeneity in a meta-analysis. STATISTICS IN MEDICINE. 2002;21(11):1539-58.

Appendix 1: Draft Search strategy

1. exp *"schizophrenia and disorders with psychotic features"/

2. (schizophren* or hebephreni* or oligophreni* or psychot* or psychosis or psychoses*).ti,ab.

3. 1 or 2

4. (recur* or reoccur* or relaps*).ti,ab.

5. (re adj3 occur*).ti,ab.

6. Recurrence/ or secondary prevention/

7. 4 or 5 or 6

8. Validat$.mp. or Predict$.ti. or Rule$.mp. or (Predict$ and (Outcome$ or Risk$ or Model$)).mp. or ((History or Variable$ or Criteria or Scor$ or Characteristic$ or Finding$ or Factor$) and (Predict$ or Model$ or Decision$ or Identif$ or Prognos$)).mp. or (Decision$.mp. and ((Model$ or Clinical$).mp. or Logistic Models/)) or (Prognostic and (History or Variable$ or Criteria or Scor$ or Characteristic$ or Finding$ or Factor$ or Model$)).mp. [mp=title, abstract, original title, name of substance word, subject heading word, keyword heading word, protocol supplementary concept word, rare disease supplementary concept word, unique identifier]

9. 3 and 6

10. 8 and 9

11. animals/ not humans/

12. exp Animals, Laboratory/

13. exp Animal Experimentation/

14. exp Models, Animal/

15. exp rodentia/

16. (rat or rats or mouse or mice).ti.

17. 11 or 12 or 13 or 14 or 15 or 16

18. 10 not 17
