# Supplementary material for: Models to predict relapse in psychosis: A systematic review
Source: PLoS One. 2017 Sep 21;12(9):e0183998. doi: 10.1371/journal.pone.0183998 (PMC5608199; doi:10.1371/journal.pone.0183998)
Supplement: S2 File — (DOCX) [file pone.0183998.s002.docx]

Search Terms

| 1. | exp *"schizophrenia and disorders with psychotic features"/ |
| --- | --- |
| 2. | (schizophren* or hebephreni* or oligophreni* or psychot* or psychosis or psychoses*).ti,ab. |
| 3. | 1 or 2 |
| 4. | (recur* or reoccur* or relaps*).ti,ab. |
| 5. | (re adj3 occur*).ti,ab. |
| 6. | Recurrence/ or secondary prevention/ |
| 7. | 4 or 5 or 6 |
| 8. | Validat$.mp. or Predict$.ti. or Rule$.mp. or (Predict$ and (Outcome$ or Risk$ or Model$)).mp. or ((History or Variable$ or Criteria or Scor$ or Characteristic$ or Finding$ or Factor$) and (Predict$ or Model$ or Decision$ or Identif$ or Prognos$)).mp. or (Decision$.mp. and ((Model$ or Clinical$).mp. or Logistic Models/)) or (Prognostic and (History or Variable$ or Criteria or Scor$ or Characteristic$ or Finding$ or Factor$ or Model$)).mp. [mp=title, abstract, original title, name of substance word, subject heading word, keyword heading word, protocol supplementary concept word, rare disease supplementary concept word, unique identifier] |
| 9. | 3 and 6 |
| 10. | 8 and 9 |
| 11. | animals/ not humans/ |
| 12. | exp Animals, Laboratory/ |
| 13. | exp Animal Experimentation/ |
| 14. | exp Models, Animal/ |
| 15. | exp rodentia/ |
| 16. | (rat or rats or mouse or mice).ti. |
| 17. | 11 or 12 or 13 or 14 or 15 or 16 |
| 18. | 10 not 17 |
